# Visual perception and visual cognition in healthy and pathological ageing

**DOI:** 10.3389/fpsyg.2014.00348

**Published:** 2014-05-06

**Authors:** Mark W. Greenlee, Allison B. Sekuler

**Affiliations:** ^1^Institute for Experimental Psychology, University of RegensburgRegensburg, Germany; ^2^Department of Psychology, McMaster UniversityHamilton, ON, Canada

**Keywords:** ageing, vision, visual cognition, central scotoma, age-related macular degeneration, Alzheimer's disease, mild cognitive impairment, functional MRI

This volume features state-of-the-art approaches to determining the effects of ageing on visual perception, visual cognition, and visually guided behavior. They incorporate psychophysics, eye movements, electrophysiology, and neuroimaging to determine how ageing affects vision in health and pathology.

Brockmole and Logie (2013) present behavioral findings on the visual working memory (VWM) abilities of over 55,000 individuals, aged 8 and 75 years, who were studied on-line to provide an analysis of age-related change in VWM. The results showed that VWM varies over the lifespan, peaking at age 20, to be followed by a sharp linear decline. By the age 55 years, adults possess poorer immediate visual memory than 8 and 9 year olds. Allard et al. (2013) present their work on age-related deficits on second-order motion processing at all temporal frequencies including the ones for which no age-related effect on first-order motion processing has so far been observed. They conclude that aging affects the ability to track moving features. Van der Stigchel et al. (2013) tested four macular degeneration (MD) patients in a visual search paradigm and contrasted their performance with that of healthy controls with and without a simulated scotoma. Saccadic search latencies for the MD group were significantly longer in both conditions compared to controls. Legault et al. (2013) report the results of a study on the capacity of older participants to improve their tracking-speed thresholds in a dynamic, virtual reality environment. Their results show that this capacity is significantly affected by healthy aging but that perceptual-cognitive training can significantly reduce age-related effects in older individuals. Bower et al. (2013) show how perceptual learning can improve motion discrimination for older, compared to younger, individuals under high- and low-contrast conditions. Both older and younger subjects exhibited lower duration thresholds after training. Graham et al. (2013) focus on patients with early Alzheimer's dementia with respect to the temporal stability of their aesthetic judgments of paintings. They find that the stability of aesthetic judgments for portrait paintings, landscape paintings, and landscape photographs is not different from those of controls, whereas the aesthetic stability for portrait photographs was significantly impaired in the AD group. Hamel et al. (2013) use a driving simulator paradigm with eye- and head-movement recordings in young and old subjects to assess age-related changes in visual exploratory behavior. No significant age effects were found regarding saccadic parameters. In the older subjects head movements increasingly contributed to gaze amplitude. Interestingly, video game-experienced subjects revealed larger saccade amplitudes and a broader distribution of fixations. They conclude that it is essential to consider video game experience in all testing methods using virtual media.

Hutchison et al. (2013) investigated the origin of age-related differences in the BOLD signal by comparing its blood flow and oxygen metabolic constituents. Using MR dual-echo arterial spin labeling and CO_2_ ingestion, they observed age-equivalent fractional cerebral blood flow (ΔCBF) in the presence of age-related increases in fractional cerebral metabolic rate of oxygen (ΔCMRO_2_). Reductions in ΔCBF responsiveness to increased ΔCMRO_2_ in elderly participants led to paradoxical age-related BOLD decreases. Age-related ΔCBF/ΔCMRO_2_ ratio decreases were associated with increases in behavioral reaction times, suggesting that age-related slowing resulted from less efficient neural activity. Alichniewicz et al. (2013) used functional magnetic resonance imaging (fMRI) to reveal the neural correlates of saccadic inhibition in young participants, in elderly participants and in patients with amnestic mild cognitive impairment (aMCI). The results indicate decreased activation in parietal lobe in healthy elderly persons compared to young persons and decreased activation in frontal eye fields in aMCI patients compared to healthy elderly persons during the execution of anti-saccades. The study by Rosengarth et al. (2013) examined the effects of oculomotor training on the fMRI response in patients with AMD. During a 6-month eccentric-fixation training period the AMD subjects and the control group took part in three functional and structural magnetic resonance imaging sessions. Oculomotor training improved fixation stability, visual acuity and reading speed. Positive correlations were evident between brain activation changes and improvements in fixation stability in the visual cortex during training. Brewer and Barton (2014) present fMRI results on visual field map (VFM) organization and population receptive fields (pRFs) between young adults and healthy aging subjects for occipital VFMs in early visual areas. Healthy aging subjects do not show major VFM organizational deficits, but do have reduced surface area and increased pRF sizes in the foveal representations of V1, V2, and hV4 relative to healthy, young controls, consistent with behavioral deficits seen in healthy aging. Results from two patients with mild Alzheimer's dementia (AD) are presented, which reveal potential changes in visual cortex.

Bieniek et al. (2013) investigated the effects of aging, luminance and individual differences on early event-related potential (ERP) components in healthy participants (aged 18–79). In two experiments they recorded EEG while participants viewed faces or noise textures at different luminance levels. They found a 1 ms per year slowdown in visual processing that was independent of luminance. Their results suggest that early ERPs to faces are delayed by aging and that these delays are of cortical, rather than optical origin. Roudaia et al. (2013a) varied contour and distracter inter-element spacing, collinearity, and stimulus duration in multiple Gabor displays. Their findings indicate that contour discrimination accuracy was lower in older subjects, but the effect of aging did not vary with contour spacing or orientation jitter. Stimulus duration had a greater effect on older subjects' performance, but only for less salient contours. Roudaia et al. (2013b) studied age-related changes in the integration of multiple inter- and intra-modal cues in the perception of moving objects that are either perceived to collide or bounce off each other. The findings indicate that a sharp sound coincident with the disks' overlap increased both groups' perception of bouncing, but did so significantly less for older subjects. The results point to a weakened inter- and intra-modal integration with aging.

It is our hope that these studies contribute to a better understanding of the effects of ageing on visual perception and visual cognition.

## Conflict of interest statement

The authors declare that the research was conducted in the absence of any commercial or financial relationships that could be construed as a potential conflict of interest.
